# Ventrolateral periaqueductal gray matter integrative system of defense and antinociception

**DOI:** 10.1007/s00424-022-02672-0

**Published:** 2022-02-24

**Authors:** Gustavo Henrique de Mello Rosa, Farhad Ullah, Yara Bezerra de Paiva, Juliana Almeida da Silva, Luiz Guilherme S. Branco, Alexandre Pinto Corrado, Priscila Medeiros, Norberto Cysne Coimbra, Audrey Franceschi Biagioni

**Affiliations:** 1 Laboratory of Neuroanatomy & Neuropsychobiology, Department of Pharmacology, Ribeirão Preto Medical School of the University of São Paulo (FMRP-USP), Av. Bandeirantes, 3900, Ribeirão Preto, São Paulo 14049-900 Brazil; 2 Department of Zoology, Islamia College Peshawar, Grand trunk Rd, Rahat Abad, Peshawar, 25120 Pakistan; 3 Department of Basic and Oral Biology, Ribeirão Preto School of Dentistry of the University of São Paulo, Av. Bandeirantes, 3900, Ribeirão Preto, São Paulo 14040-904 Brazil; 4 Laboratory of Neurosciences of Pain & Emotions and Multi-User Centre of Neuroelectrophysiology, Department of Surgery and Anatomy, Ribeirão Preto Medical School of the University of São Paulo, Av. Bandeirantes, 3900, Ribeirão Preto, São Paulo 14049-900 Brazil; 5 Behavioural Neuroscience Institute (INeC), Av. do Café, 2450, Ribeirão Preto, São Paulo 14050-220 Brazil; 6 Neuron Physiology and Technology Laboratory, International School for Advanced Studies (SISSA), Department of Neuroscience, Via Bonomea 265, 34136 Trieste, Italy

**Keywords:** Defensive immobility, Antinociception, Respiratory function, Ventrolateral column of periaqueductal gray matter

## Abstract

Defensive responses are neurophysiological processes crucial for survival during threatening situations. Defensive immobility is a common adaptive response, in rodents, elaborated by ventrolateral periaqueductal gray matter (vlPAG) when threat is unavoidable. It is associated with somatosensory and autonomic reactions such as alteration in the sensation of pain and rate of respiration. In this study, defensive immobility was assessed by chemical stimulation of vlPAG with different doses of NMDA (0.1, 0.3, and 0.6 nmol). After elicitation of defensive immobility, antinociceptive and respiratory response tests were also performed. Results revealed that defensive immobility was followed by a decrease in the nociceptive perception. Furthermore, the lowest dose of NMDA induced antinociceptive response without eliciting defensive immobility. During defensive immobility, respiratory responses were also disturbed. Interestingly, respiratory rate was increased and interspersed with prolonged expiratory phase of breathing. These findings suggest that vlPAG integrates three different defensive behavioral responses, contributing to the most effective defensive strategies during threatening situations.

## Introduction

Threatening situations are known to influence defensive behavioral reaction patterns, causing motor and autonomic response expressions to enhance the survival probabilities [14, 20]. Ethological studies emphasizing unconditioned fear showed that different threatening contexts elicit different defensive behavioral strategies [5, 11]. For instance, unavoidable fear stimuli lead to immobility defensive reaction in rodents, characterized by a tense posture and entire body immobility, except breathing movement which can be visualized [6, 9]. The defensive state has been reported to be organized by the ventrolateral periaqueductal gray matter (vlPAG) column [4, 51]. The same nuclei have been also reported to integrate autonomic and sensory information that accompanies defensive behavior [29, 32, 49]. Defensive immobility organized by the vlPAG neurons have been described during several conditions such as contextual fear conditioning [8, 45], prey versus predator paradigm [10], and midbrain tectum electrical and chemical stimulation [47, 48]. Despite the well-reported pre-escape and after-escape defensive immobility elaborated by the periaqueductal gray matter (PAG), the associative somatosensory and autonomic reactions such as pain sensation and respiratory rate organized by vlPAG are not well known yet.

Enhanced sensory processing is suggested to optimize defensive strategy during an inescapable predator encounter [19, 46]. When a prey faces a threat, the neural circuits of fear inhibit the somatosensory system, resulting in defensive behavior and antinociception [27, 30, 31]. After antinociceptive pathways are activated, the injured prey is able to focus on defensive behavior performance instead of pain-related recuperative behavior, which increases the chances of survival [7, 46]. During inescapable threat, the integrated defensive immobility and antinociceptive responses produced by the activation of the vlPAG neurons were observed to occur simultaneously [29]. However, some studies suggest that there is no pharmacological relationship between these two responses. It has been examined that defensive antinociception can be reversed by opioid and serotoninergic antagonist local treatments; however, these treatments showed no effect on the defensive behavioral responses elaborated by the vlPAG neurons [16]. In order to determine whether defensive immobility and defensive antinociception are mutually dependent or not, we treated vlPAG with different doses of N-methyl-D-aspartic acid (NMDA) and examined its effect in eliciting defensive immobility and antinociceptive responses.

Furthermore, the contribution of vlPAG in respiratory control associated with defensive strategies was also currently analyzed. In fact, it has been proposed that PAG may link both defensive immobility and changes in breathing rate [44]. Here, we examined the pattern of defensive immobility behavior produced by vlPAG after its chemical stimulation and its relationship with defensive antinociception and respiratory processes, which are crucial for the survival of prey during threating situations.

## Methods

### Ethical approval

The experiments were carried out according to the ethical principles developed by the Ethics Committee on Animal Experimentation of FMRP-USP (CETEA), which are in accordance with the ethical principles National Commission on Ethics in Animal Experimentation (CONCEA) protocol on registration 96/2010.

## Animals

Male Wistar rats (*Rattus norvegicus*, Rodentia, Muridae), weighing 250–300 g (*n* = 8 per group), were used in these behavioral experiments. The enclosure was maintained at 21 ± 2 °C on a light–dark cycle. Lights were on from 7 a.m. to 7 p.m. Animals were housed in groups of four in acrylic cages (41 × 34 cm) and were given free access to food and water throughout the experiment. Psychobiological experiments were performed between 9 a.m. and 4 p.m. Surgeries of all rats were performed under deep anesthesia. Every experimental procedure was planned to minimize the suffering of animals and to decrease the number of animals used in the experiment.

## Drugs

N-methyl-D-aspartic acid (NMDA; Sigma-Aldrich, St. Louis, USA) was used to stimulate the midbrain. Different doses of NMDA (0.1, 0.3, and 0.6 nmol) were injected in the vlPAG. The vehicle (0.9% NaCl) was also used as a control. All injections were performed in a volume of 0.2 µL.

## Stereotaxic surgery

The animals were deeply anaesthetized with intramuscular injections of ketamine in a dose of 100 mg/kg and xylazine in a dose of 10 mg/kg. After anesthesia, the head of each animal was fixed in a stereotaxic frame (David Kopf, Tujunga, CA, USA). Stainless steel guide cannulas were implanted in the midbrain 1 mm above the vlPAG with the following coordinates, considering bregma as a reference: antero-posterior =  − 7.8 mm, middle-lateral = 0.8 mm, and dorso-ventral = 6.4 mm, in an angle of 20°, according to the rat brain in stereotaxic coordinate atlas [38]. Stereotaxic placement of guide cannulas was angled to avoid damage and bleeding from the superior sagittal sinus [17]. Five days later, rats received a random treatment into vlPAG of either physiological saline or NMDA delivered by a needle (0.3 mm of outer diameter) linked to a syringe (Hamilton) through a polyethylene tube. The microinjection needle was inserted through the guide-cannula until it reached the vlPAG (1 mm below the guide-cannula). Injections occurred over a 40-s period. Only rats that received microinjection within the vlPAG were included in the present study. At the end of the surgery, each animal received an intramuscular injection of benzathine penicillin G (120,000 IU; 0.1 mL) and a subcutaneous injection of the non-steroidal analgesic and anti-inflammatory meglumine flunixin (2.5 mg/kg).

## Experimental procedures

### Behavioral testing

The behavioral tests were carried out in a circular arena (50 cm wide × 60 cm high) with transparent acrylic wall located in a lighted experimental room (350 lx at the arena floor level). The circular arena floor was divided into 12 equal sections used to count crossings. Defensive immobility behavior was defined as absence of body movements for 6 s, except respiratory movements. The frequency and duration of this behavior was recorded, during 5 min, immediately after rats received a single microinjection of either physiological saline or NMDA in the mesencephalic tegmentum.

### Antinociceptive testing

The rats had their nociception thresholds compared using the tail-flick test. Each animal was placed in a restraining apparatus (Insight, Ribeirão Preto, Brazil) with acrylic walls, and its tail was placed on a heating coil (tail-flick Analgesia Instrument; Insight, Ribeirão Preto, Brazil). The amount of heat applied to the tail was increased until the animal removed its tail from the apparatus. The coil (Ni/Cr alloy; 26.04 cm in length × 0.02 cm in diameter) began at room temperature (approximately 20 °C), and then current was applied to increase the temperature of the coil at a rate of 9 °C/s [2, 15, 18, 28]. If necessary, small adjustments were made to the intensity of the current at the beginning of the experiment to obtain three consecutive tail-flick latencies (TFL) between 2.5 and 3.5 s. If the animal did not remove its tail from the heater within 6 s, the apparatus was turned off to prevent damage to the skin. Three baseline measurements of control TFL were taken at 5-min intervals. TFL measured every 5 min for 30 min (1st, 2nd, 3rd, 4th, 5th, 6th, and 7th tail withdrawal measurement) were taken 1 min after end of the defensive behavior assay.

### Measurements of pulmonary ventilation

Respiratory frequency (*f*_*R*_), tidal volume (*V*_*T*_), and consequently ventilation (*V*_*E*_) were obtained using the barometric method (whole-body plethysmography (WBP)) according to Bartlett and Tenney [1] and Malan [33] and used in previous studies [40, 41]. In brief, rats were housed fully conscious and unrestrained within the WBP (manufactured by Precision Workshops Section, University of São Paulo, Ribeirão Preto) chamber, which consist of a plexiglas square box (30 × 30 × 30 cm). Chamber was used in a closed mode to allow the WBP assessment. Therefore, the inlet and the outlet of the chamber were closed by a luer lock connector or a three-position valve, respectively. Air flow was stopped and the chamber remained completely sealed during the performance of each ventilation measurement. The calibration volume was performed with a known volume of air (1 mL) injected by an insulin syringe into the chamber. Respiratory functions were estimated considering the chamber pressure changes which were captured by a pressure transducer and digitization (PowerLab DAQ) was performed with an analog–digital interface (PowerLab 8/35; ADInstruments) and LabChart software (ADInstruments). Two respiratory variables were measured, *f*_*R*_ and *V*_*T*_, the last calculated through the formula: *V*_*T*_ = *P*_*T*_/*P*_*K*_ × *V*_*K*_ × *T*_*C*_/*T*_*R*_ × (*P*_*B*_ - *P*_*C*_)/(*P*_*B*_ - *P*_*C*_) - *T*_*C*_/*T*_*b*_ x (*P*_*B*_ − *P*_*R*_), where *V*_*K*_ is the calibration volume, *P*_*T*_ is the pressure deflection associated with each volume of tidal air, *P*_*K*_ is the pressure deflection associated with each volume of air injected for calibration, and *T*_*b*_ is the body temperature. Also, *T*_*R*_ is the room temperature, *T*_*C*_ is the air temperature inside the chamber, *P*_*B*_ is the pressure barometric, *P*_*C*_ is the water vapor pressure at body temperature, and *P*_*A*_ is the pressure of water vapor at chamber temperature. *V*_*E*_ was measured by the product of *f*_*R*_ by *V*_*T*_. Body temperature (*T*_*b*_) was measured using temperature sensors (data loggers; DLs; SubCue) implanted intraperitoneally, on the same day of the stereotaxic surgery. DLs were previously programmed to acquire data every 5 min. The DLs were removed and the data were downloaded using specific software (SubCue, Calgary, Canada).

### Experimental design

Animals were submitted to surgical procedure to implant a guide cannula targeting the vlPAG. Rats were allowed to recover from surgery for a period of 5 days. The experiment consisted of 2 procedures, on successive days. First day, nociceptive thresholds of rats were measured to determine the baseline after a habituation period of 10 min. Then, rats were gently wrapped in a cloth and hand held while they received a random treatment into the vlPAG of either physiological saline or a single dose of NMDA. Immediately after the microinjection procedure, rats were placed in the open field apparatus and behavioral response was recorded during 5 min. After 1 min, nociceptive responses (TFL) were measured every 5 min during 30 min. Second day, the pulmonary ventilation was evaluated in the whole-body plethysmograph chamber in the same group of animals. After a habituation period of 30 min, three baseline measurements of pulmonary function were recorded at 5-min intervals. Subsequently, rats were submitted again to the microinjection procedure while they were inside the plethysmography chamber. Animals received the same treatment of the day before that is either saline or the same dose of NMDA. Immediately after, pulmonary measurements were evaluated during 5 min with an interval of 1 min. During the interval, the chamber was kept open for the entry of atmospheric air. For each respiratory function recording, events were collected and an average of 20-s recording was analyzed starting immediately after the microinjection, and other two recording events with the same events pattern were analyzed (Phase 1). Respiratory function was notably interspersed with episodes of expansion of expiratory time, starting approximately 40 s after the microinjection (Phase 2). Recording events from those episodes were also calculated as average of 20-s recording.

Independent groups of rats were also submitted to stereotaxic surgical procedure to implant a guide cannula targeting the vlPAG. Five days later, nociceptive thresholds of rats were measured to determine the baseline a habituation period of 10 min. Then, rats were gently wrapped in a cloth and hand held while they received a random treatment into the vlPAG of either physiological saline or 0.1 µL of NMDA. One minute later, nociceptive responses (TFL) were measured every 5 min during 30 min.

### Histology

Upon completion of the experiments, each animal was anaesthetized with ketamine at 92 mg/kg (Ketamina®) and xylazine at 9.2 mg/kg (Dopaser®) and perfused through the left cardiac ventricle using an infusion pump (Master Flex® L/S TM, Vernon Hills, IL, USA). The thoracic descending aorta was clamped, the pericardium was removed to allow perfusion through left ventricle, and the blood was washed out with Tyrode’s solution (40 mL at 4 °C). The animal was then perfused with 200 mL ice-cold 4% (w/v) paraformaldehyde in 0.1 M sodium phosphate buffer (pH 7.3) for 15 min at a pressure of 50 mmHg. The encephalon was quickly removed and maintained in 4% paraformaldehyde for at least 4 h and was then immersed in a 10% sucrose solution for 48 h. Tissue pieces were immersed in 2-methylbutane (Sigma-Aldrich, St. Louis, USA), frozen on dry ice (30 min), embedded in Tissue-Tek, and cut on a cryostat (CM 1950, Leica, Mannheim, Germany). Slices were then mounted on glass slides that were coated with chrome alum gelatin to prevent detachment and stained in a robotic Autostainer (CV 5030 Leica Autostainer) with hematoxylin–eosin. The sections were viewed under a motorized photomicroscope (AxioImager Z1, Zeiss, Oberkochen, Germany), and the positions of the tips of the guide cannula were localized according to Paxinos and Watson’s stereotaxic atlas [38].

## Statistical analysis

Data from independent groups of animals submitted to the vlPAG chemical stimulation were checked for normality and homogeneity. Behavioral responses were analyzed using one-way analysis of variance followed by Newman-Keuls posthoc test. For the tail-flick latencies, a repeated measures two-way ANOVA was applied to evaluate the statistical distribution of the data sets. Respiratory response (*f*_*R*_, *V*_*T*_, and *V*_*E*_) were evaluated by repeated measures two-way ANOVA followed by Bonferroni post hoc test. All values were expressed as mean ± SEM. The *p* values < 0.05 were considered statistically significant.

## Results

**Fig. 1:**
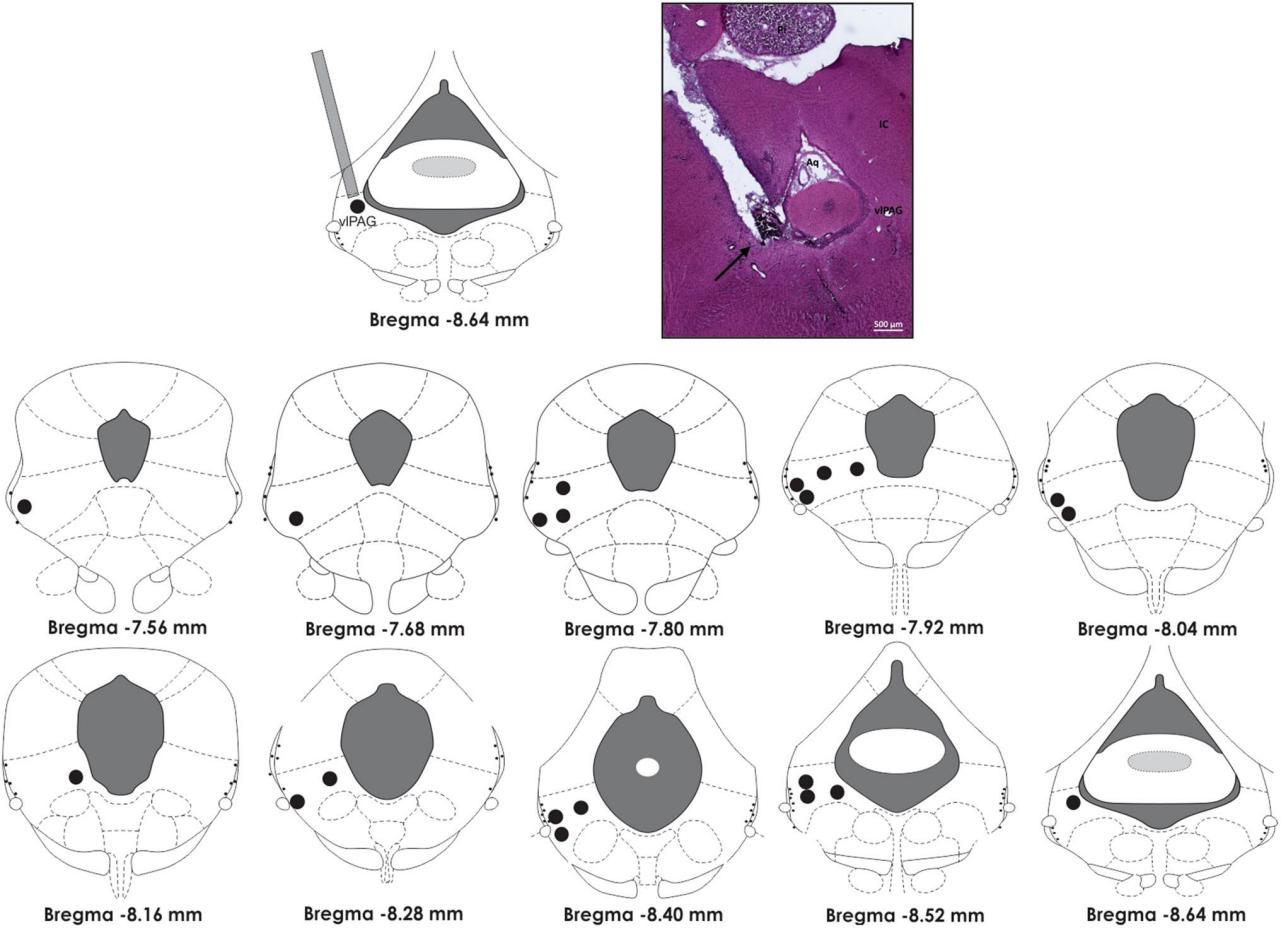
Schematic view (in the top left corner) of a guide-cannula inserted in the ventrolateral column of the periaqueductal gray matter (vlPAG). A photomicrograph of a representative site (black arrow) of microinjections of drugs in vlPAG is provided in the top right corner. In the bottom, drawings of midbrain transverse sections across rostrocaudal extensions of the periaqueductal gray matter, depicting the representation of the histologically confirmed injection sites (black circles) of either physiological saline or NMDA in the vlPAG. The number of points in the figure is fewer than the total number of rats because of overlapping injection sites. *vlPAG* ventrolateral columns of the periaqueductal gray matter, *Pi* pineal gland, *Aq* aquaeductus Sylvii, *IC* inferior colliculus. Calibration bar: 500 µm

Midbrain tegmentum chemical stimulation performed with NMDA microinjection in vlPAG induced the defensive behavioral response of defensive immobility, alteration in the nociceptive threshold, and pulmonary ventilation. The position of the cannula was histologically confirmed, and needle tracts were found between bregma − 7.56 mm and − 8.64 mm (Fig. 1). Small variations in the guide-cannula position were found; therefore, only rats with cannula tracks ending 1 mm above the vlPAG area were included in the study.

**Fig. 2:**
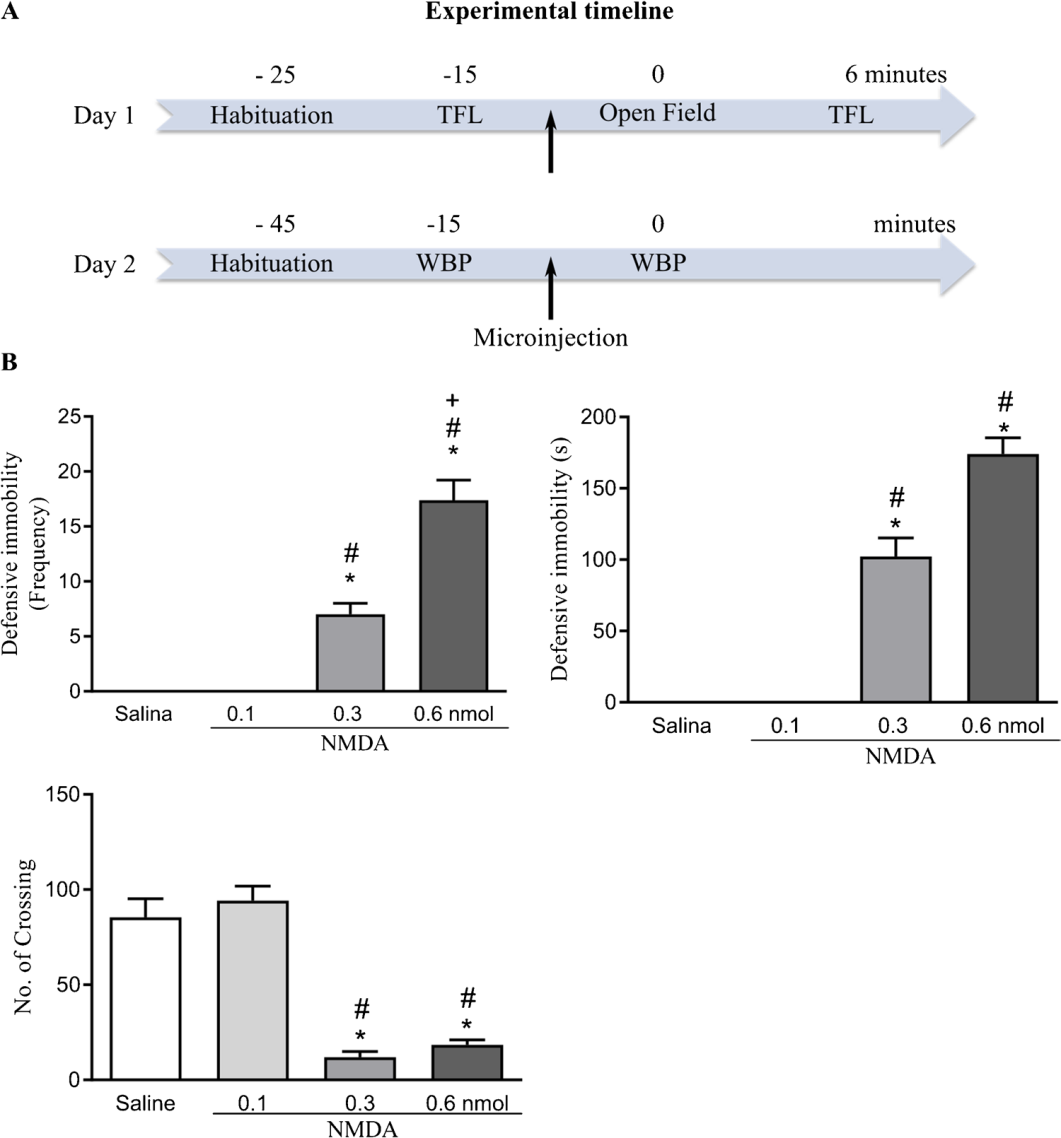
**A** Schematic representation of experimental timeline showing the sequence of the experimental procedures, in minutes. Day 1, rats were submitted to restraint habituation and tail-flick latency (TFL) baseline test, followed by exposure to open field apparatus and subsequent TFL assessment. Day 2, rats were habituated to the plethysmography chamber and whole-body plethysmography measures were recording for baseline and post-injection analysis. Arrows indicate the microinjection procedure. **B** Effect of chemical stimulation of vlPAG with microinjection of NMDA, at doses of 0.1, 0.3, and 0.6 nmol, or physiological saline on the behavioral response of defensive immobility (top) and locomotor activity expressed by the number (no.) of crossing (bottom). *p* < 0.001, compared with the control group (saline); ^#^*p* < 0.001, when compared to the group treated with 0.1 nmol NMDA according to the one-way analysis of variance (one-way ANOVA), followed by the Newman-Keuls post hoc test. The columns represent the mean ± the standard error of the mean. *N* = 8. Frequency: number of times that rats expressed defensive immobility response. s: seconds

The vlPAG treatment with NMDA at 0.3 nmol and 0.6 nmol caused increase in the frequency [*F* (3,28) = 61.45; *p* < 0.001] and in the duration [*F* (3,28) = 98.24; *p* < 0.001] of defensive immobility response compared to the groups treated with physiological saline and NMDA at the lowest dose (0.1 nmol), as shown in Fig. 2. The effect of NMDA treatment was also observed in the locomotor activity expressed by the number of crossing [*F* (3,28) = 43.30; *p* < 0.001] compared to the groups treated with physiological saline and NMDA at 0.1 nmol (Fig. 2).

**Fig. 3:**
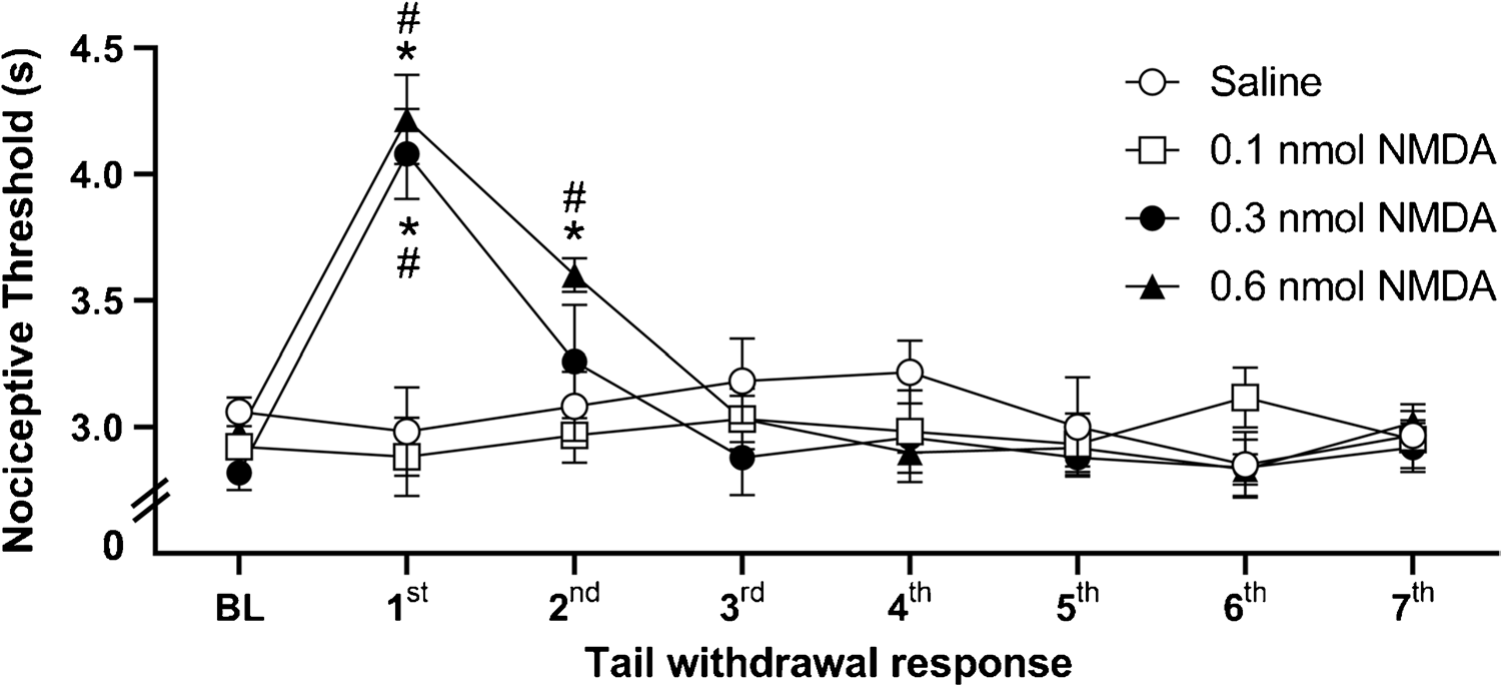
Effect of chemical stimulation of vlPAG with microinjection of NMDA, at doses of 0.1, 0.3, and 0.6 nmol, or of physiological saline on the nociceptive threshold. *N* = 8. *p < 0.001 when compared to the control group (saline); ^#^*p* < 0.001 compared to NMDA at a dose of 0.1 nmol, according to the repeated measures analysis of variance, followed by Duncan’s post hoc test. BL: baseline tail withdrawal test, measured before treatment. *X*-axis: tail withdrawal response consecutive measurement taken at intervals of 5 min. *Y*-axis: nociceptive threshold (measured in s). Data were represented as means ± SEM

Defensive immobility elicited by chemical stimulation of vlPAG with NMDA was followed by defensive antinociception recorded during 5 min after the defensive response. According to the repeated measures two-way ANOVA, there was a statistically significant effect of treatment [*F* (3,28) = 5.6; *p* < 0.01], of time [*F* (7,22) = 9.34; *p* < 0.001], and of treatment versus time interaction [*F* (21,62) = 7.21; *p* < 0.001]. The vlPAG chemical stimulation with NMDA at 0.3 nmol and 0.6 nmol increased the tail-flick latencies [*F* (3,28) = 12.75; *p* < 0.001] in comparison to the control group and to the group treated with NMDA at the lowest dose of 0.1 nmol (Fig. 3).

**Fig. 4:**
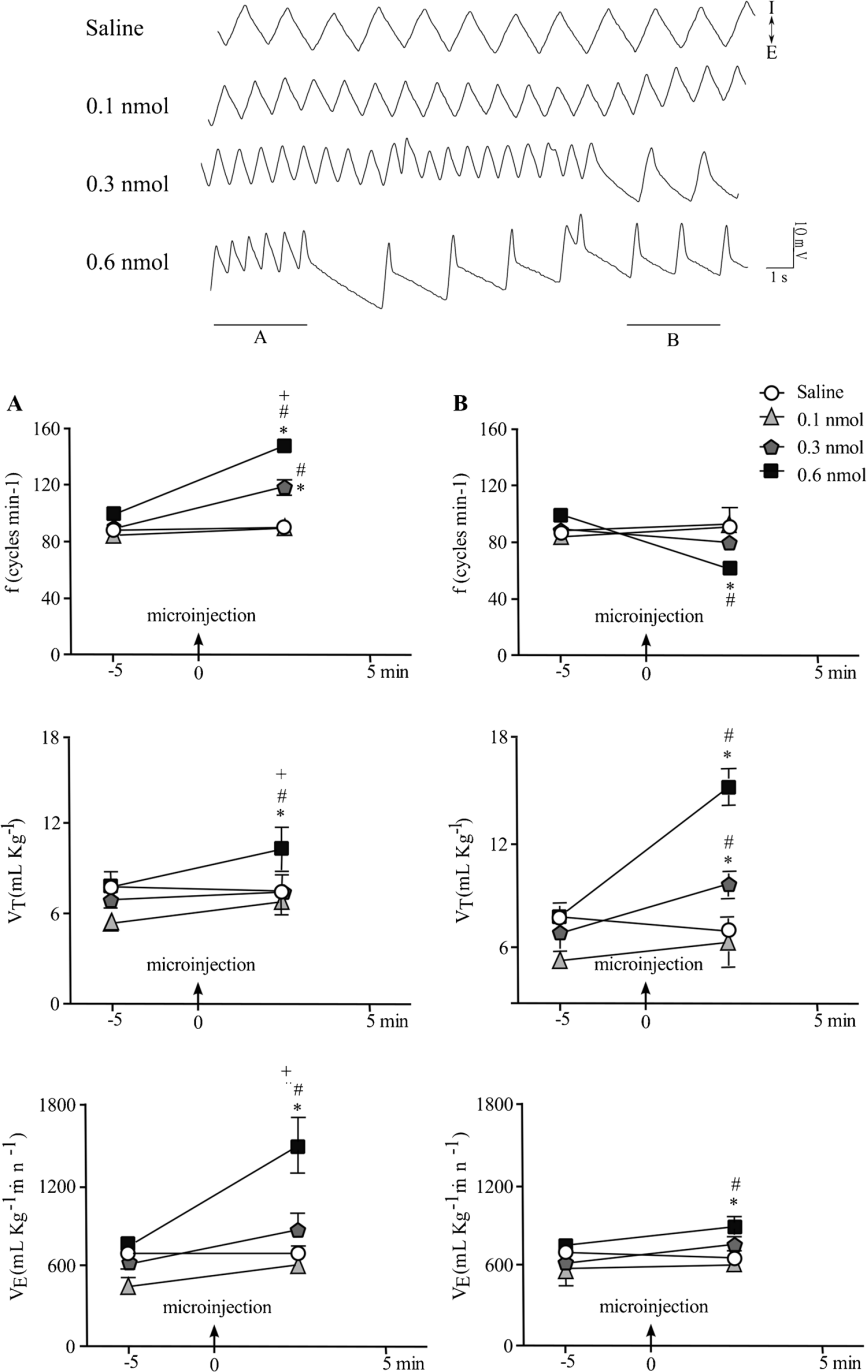
Representative recording (top) and bar plots (bottom) of respiratory responses showing the effect of the vlPAG treatment with either physiological salina or NMDA (0.1, 0.3, or 0.6 nmol) in the *f*_*R*_, *V*_*T*_, and *V*_*E*_ assessed during 5 min starting immediately after the microinjection (**A**) and during the interspersed episodes of expansion of expiratory time (**B**). *p < 0.001, compared with the control group (saline); ^#^*p* < 0.001 compared to the group treated with NMDA 0.1 nmol; ^+^*p* < 0.01 compared to 0.3-nmol-NMDA treated group. The columns represent the mean ± the standard error of the mean. *N* = 7–8. *f*_*R*_ respiratory frequency, *V*_*T*_ tidal volume, *V*_*E*_ minute ventilation

**Fig. 5:**
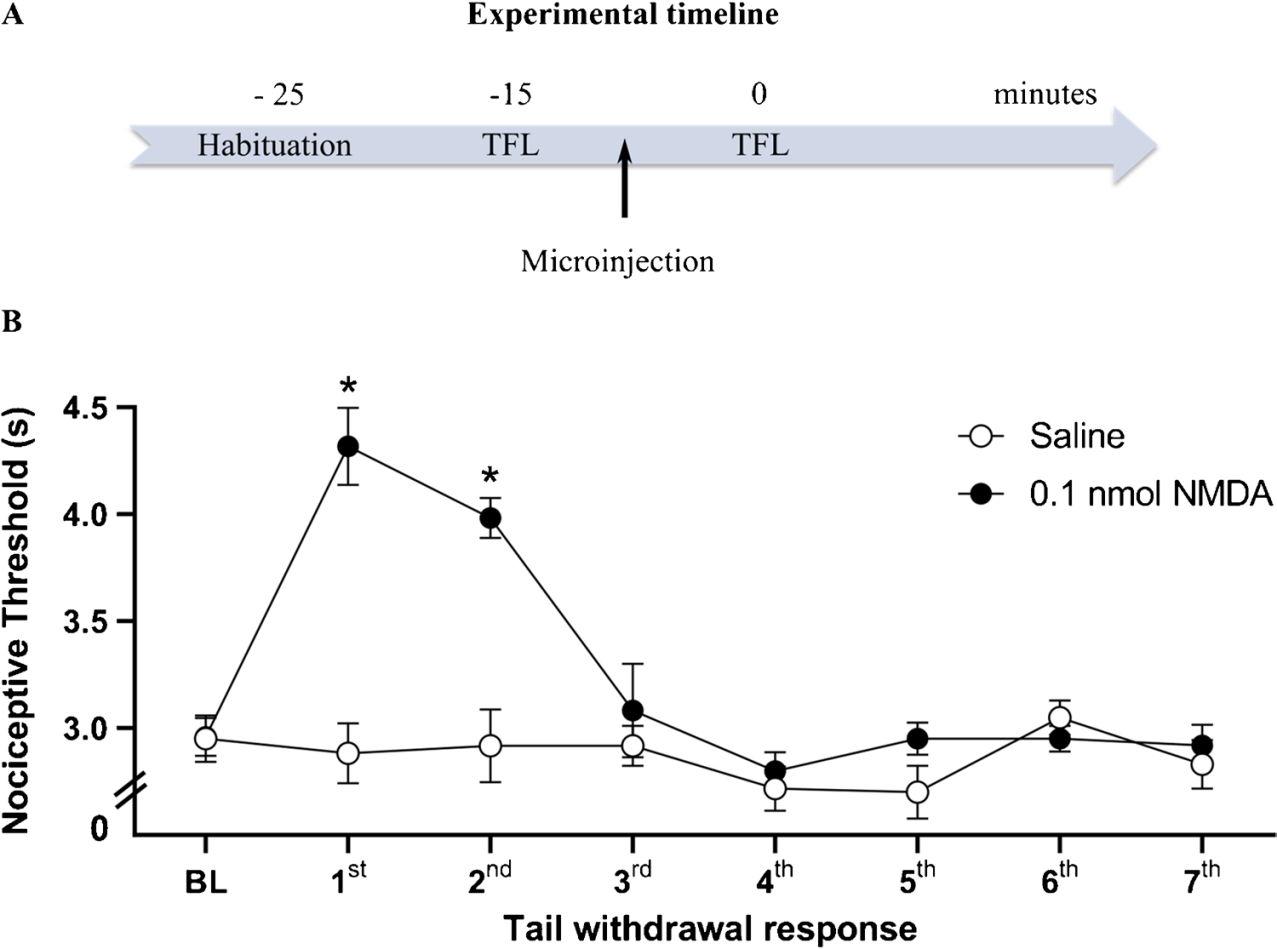
**A** Schematic representation of experimental timeline showing the sequence of the experimental procedures of restraint habituation and tail-flick latency (TFL) test in minutes. Arrow indicates the microinjection procedure. **B** Effect of chemical stimulation of vlPAG with microinjection of 0.1 nmol NMDA or saline on the nociceptive threshold. *N* = 8. *p < 0.001 when compared to the control group (saline), according to the repeated measures ANOVA, followed by Duncan’s post hoc test. BL: baseline tail withdrawal test, measured before treatment. *X*-axis: tail withdrawal response consecutive measurement taken at intervals of 5 min. *Y*-axis: nociceptive threshold (measured in s). Data were represented as mean ± SEM

The microinjection of NMDA in vlPAG caused a dynamic change in respiratory responses characterized by a period of increase in the respiratory frequency (Phase 1; Fig. 4A) interspersed with episodes of prolonged expiratory phase of breathing (Phase 2; Fig. 4B). There was a statistically significant effect of treatment [*F* (1,27) = 127.8; *p* < 0.001 and *F* (1,27) = 15.46; *p* < 0.001], time [*F* (3,27) = 33.86; *p* < 0.001], and treatment versus time interaction [*F* (3,27) = 32.91; *p* < 0.001 and *F* (3,27) = 21.7; *p* < 0.001] on *f*_*R*_, regarding the Phase 1 and 2, respectively. For *V*_*T*_ there was a statistically significant effect on treatment [*F* (1,27) = 7.69; *p* < 0.01 and *F* (1,27) = 60.88; *p* < 0.001], time [*F* (3,27) = 7.23; *p* < 0.01 and *F* (3,27) = 39.56; *p* < 0.001], and the treatment versus time interaction [*F* (3,27) = 2.7; *p* > 0.05 and *F* (3,27) = 26.16; *p* < 0.001] regarding the Phase 1 and 2, respectively. Considering the *V*_*E*_, there was a statistically significant effect of treatment [*F* (1,27) = 30.34; *p* < 0.001 and *F* (1,27) = 5.38; *p* < 0.05], of time [*F* (3,27) = 20.97; *p* < 0.001 and (*F* (3,27) = 6.27; *p* < 0.01], and of treatment versus time interaction [*F* (3,27) = 9.18; *p* < 0.001 and *F* (3,27) = 2.3; *p* > 0.05] in the Phase 1 and 2, respectively. vlPAG activation caused an initial increase in the *f*_*R*_, *V*_*T*_, and *V*_*E*_ after the intramesencephalic injection of NMDA in a dose of 0.6 nmol (*p* < 0.001) compared to all other groups (Fig. 5A). Those responses were interspersed with a decrease in the *f*_*R*_ (*p* < 0.001) and prolonged expiratory phase increasing the *V*_*T*_ (*p* < 0.001) and *V*_*E*_ (*p* < 0.001) compared to control group, and to NMDA (0.1 nmol and 0.3 nmol)-treated groups (Fig. 4B). vlPAG treatment with NMDA at 0.3 nmol also caused a dynamic change in respiratory responses with a significant increase in the *f*_*R*_ (*p* < 0.001) with no significant effect on *V*_*T*_ and *V*_*E*_ (Phase 1), followed by increase in the *V*_*T*_ (*p* < 0.001) and without changes in the basal *f*_*R*_ and *V*_*E*_ compared to control and to vlPAG stimulation with NMDA in a dose of 0.1 nmol (Fig. 4 A and B, respectively).

In order to evaluate the effect of small dose of NMDA in the nociceptive threshold, independent groups of animals were submitted to the tail-flick test immediately after the intramesencephalic microinjection of the excitatory amino acid. vlPAG chemical stimulation elicited antinociception up to 5 min, which is in line with time of effect of NMDA at 0.3 and 0.6 evaluated in behavioral and respiratory responses. According to the repeated measures two-way ANOVA, there was significant effects of treatment [*F* (1.14) = 24.55; *p* < 0.001], of time [*F* (7.8) = 16.55; *p* < 0.001], and of treatment versus time interaction [*F* (7.8) = 11.72; *p* < 0.001]. Chemical stimulation of vlPAG with NMDA at the lowest dose (0.1 nmol) increased the tail-flick latencies [*F* (1.14) = 50.4; *p* < 0.001] when compared to the physiological saline-treated control group (Fig. 5).

## Discussion

Defensive immobility response, characterized by cessation of voluntary movements, was observed in the present study after the NMDA microinjection in the vlPAG. The pronounced effects of electrical and chemical stimulations of the ventrolateral portion of PAG in eliciting immobility are well established [4, 34, 48, 50]. Our findings indicate that vlPAG neuronal circuits integrate defensive behavioral responses, sensory, and autonomic reactions. Consistently with a previous report [4], we demonstrated that the chemical stimulation of this mesencephalic region induces a defensive immobility response in a dose-dependent manner. Moreover, the activation of the excitatory glutamatergic receptors of the vlPAG also inhibits pain perception and elicits respiratory changes, representing the unconditioned fear-induced antinociception displayed by laboratory animals and also chemical stimulation of dorsal columns of the PAG [13], and by those threatened by a natural predator [14].

We also observed that intermediate and high doses of NMDA microinjected into vlPAG significantly increased the nociceptive threshold, evaluated after the defensive immobility response. The integrative systems of defensive behavior and attenuation of pain-related responses lead to the optimal strategy for survival [14]. The perceptual-defensive-recuperative theory proposed by Bolles and Fanselow [7] suggests that fear inhibits pain, facilitating the perception of danger instead of recuperative responses that might compromise the animal survival. Interestingly, the treatment of vlPAG with low dose of NMDA activated antinociceptive process without eliciting defensive immobility response. It suggests that vlPAG-elaborated antinociception and behavioral responses can occur independently. We can only speculate that antinociceptive process, observed promptly after NMDA (0.1 nmol) vlPAG treatment, is induced by the local action of the NMDA in the vlPAG neurons. On the other hand, the antinociception that follows the behavioral defensive responses may be related to the activation of the entire circuit underlying fear [3, 14]. In fact, time of effect of NMDA into the vlPAG lasts approximately 5 min [36], as observed in the tail-flick test immediately after the injections of 0.1 nmol NMDA and after 0.3- and 0.6-nmol treatment evaluated in behavioral and respiratory responses. Altogether, these results are consistent with the hypothesis that nociceptive reflexes and immobility may not occur simultaneously [35]. Despite the fact that both freezing and antinociception are coordinated by vlPAG GABA-benzodiazepine mechanisms [16], distinct neuronal pathway of the ventrolateral periaqueductal gray is likely to contribute to fear-related defensive behavior, integrating sensory and motor mechanisms.

In addition to the well-evidenced behavioral repertoire organized by vlPAG, it was observed in the present study that chemical stimulation of vlPAG induced irregular breathing pattern. The caudal part of vlPAG plays key role in controlling the spontaneous breathing pattern [43] whose activation triggers dysfunctional reactions related to anxiety disorder [25]. In the present study, we observed dysfunction in the respiratory responses during the expression of defensive immobility. The increased respiratory frequency and pulmonary ventilation were interspersed with episodes of reduction in respiratory frequency and prolonged expiratory phase of breathing, which is considered an adaptive response crucial for defensive immobility performance when a threat is imminent [39]. It is previously suggested that vlPAG stimulation evokes irregular tachypnea characterized by increase in the respiratory frequency followed by its decrease along with episodes of apnea [42–44]. Despite the fact that we did not observe apnea in this study, our results are in line with evidence suggesting that vlPAG coordinates somatomotor mechanisms that are required in the context of basic survival [12]. Indeed, midbrain structures are activated in rodents, threatened by venomous coral snakes in the polygonal arena for snake test [37]. The irregular respiratory responses organized by vlPAG neurons may be under the influence of dPAG [26] and lPAG descending projections [43]. In fact, the different columns of PAG have been reported to regulate several respiratory patterns. While dorsal region stimulation causes tachypnea [42], the lateral column promotes inspiratory apneusis and caudal ventrolateral PAG induces irregular breathing in cats [44]. In addition, vlPAG maintains connections with the limbic system and cerebral cortical structures that are crucial for regulating the behavioral response and respiratory functions to promote survival [21, 24]. Respiratory rate is particularly coordinated by the different columns of PAG during the threating stimulus [22]. It was demonstrated through functional magnetic resonance in humans that PAG lateral columns are activated during conditioned respiratory threat test. On the other hand, the PAG ventrolateral column was activated in the anticipatory phase [22]. In the same study, it is suggested that lateral columns of PAG modulate sensorimotor response during shortness of breath, and the PAG ventrolateral columns participate in the perception of the threat, preventing shortness of breath [42]. Thus, different columns of PAG modulate eupnea according to specific behavioral responses during dangerous situations.

## Conclusion

The current study provided novel insights on the organization of such defensive responses that depend on the intensity of the stimulus. In this sense, highest tested dose of NMDA induced defensive immobility behavior, fear-induced antinociception, and tachypnea in a dose-dependent manner. On the other hand, the lowest dose of NMDA was only effective to induce defensive antinociception, probably because the antinociceptive process occurs in a dissociated manner recruiting different neuronal circuits, such as opioid and serotonergic mechanisms [16]. Furthermore, vlPAG coordinates the functional correlation between breathing pattern and fear-related behavioral responses, which are essential for survival. This neuronal circuit may constitute a key limbic system-related component, linking respiratory signals with behavioral responses to threat [23], which is highly correlated to anxiety disorders. Thus, vlPAG is a potential target structure for the treatment of anxiety disorder related to breathing pattern abnormalities.

## Data Availability

Data will be made available on request.

## Abbreviations

vlPAG: Ventrolateral column of periaqueductal gray matter
